# Plant growth-promoting effect and genomic analysis of the *P. putida* LWPZF isolated from *C. japonicum* rhizosphere

**DOI:** 10.1186/s13568-022-01445-3

**Published:** 2022-08-02

**Authors:** Tingting Jin, Jiahong Ren, Yunling Li, Bianxia Bai, Ruixiang Liu, Ying Wang

**Affiliations:** Department of Life Sciences, Changzhi University, Changzhi, 046011 People’s Republic of China

**Keywords:** Plant growth promotion, *Pseudomonas putida*, Genome analysis, Heavy metal resistance, Antifungal activity

## Abstract

Plant growth-promoting rhizobacteria are a type of beneficial bacteria which inhabit in the rhizosphere and possess the abilities to promote plant growth. *Pseudomonas putida* LWPZF is a plant growth-promoting bacterium isolated from the rhizosphere soil of *Cercidiphyllum japonicum*. Inoculation treatment with LWPZF could significantly promote the growth of *C. japonicum* seedlings. *P. putida* LWPZF has a variety of plant growth-promoting properties, including the ability to solubilize phosphate, synthesize ACC deaminase and IAA. The *P. putida* LWPZF genome contained a circular chromosome (6,259,530 bp) and a circular plasmid (160,969 bp) with G+C contents of 61.75% and 58.25%, respectively. There were 5632 and 169 predicted protein-coding sequences (CDSs) on the chromosome and the plasmid respectively. Genome sequence analysis revealed lots of genes associated with biosynthesis of IAA, pyoverdine, ACC deaminase, trehalose, volatiles acetoin and 2,3-butanediol, 4-hydroxybenzoate, as well as gluconic acid contributing phosphate solubilization. Additionally, we identified many heavy metal resistance genes, including arsenate, copper, chromate, cobalt-zinc-cadmium, and mercury. These results suggest that *P. putida* LWPZF shows strong potential in the fields of biofertilizer, biocontrol and heavy metal contamination soil remediation. The data presented in this study will allow us to better understand the mechanisms of plant growth promotion, biocontrol, and anti-heavy metal of *P. putida* LWPZF.

**Supplementary Information:**

The online version contains supplementary material available at 10.1186/s13568-022-01445-3.

## Introduction

Plant growth-promoting rhizobacteria (PGPR) are a type of beneficial bacteria inhabited in the rhizosphere (Gouda et al. 2018; Lugtenberg and Kamilova 2009). PGPR develop various strategies to facilitate plant growth. These mechanisms include phosphate solubilization (Alori et al. 2017), nitrogen fixation (Pham et al. 2017), indole-3-acetic acid (IAA) secretion (Spaepen et al. 2007), siderophores production (Miethke and Marahiel 2007), 1-amino-cyclopropane-1-carboxylate deaminase (ACCD) (Singh et al. 2015) and antibiotics (Liu et al. 2018) synthesis. Many PGPR have the ability of heavy metal (HM) resistance, which can reduce HM availability and plant uptake of HMs (Cheng et al. 2021; Xu et al. 2017). In comparison to the decline in soil fertility and environmental pollution caused by chemical fertilizer use, PGPRs have come to be considered as new inoculants in the area of biofertilizer technology owing to their diverse plant growth-promoting (PGP) properties and environmental friendliness (Bhattacharyya and Jha 2012).

Systematic analysis of whole genome has enabled us to understand the growth-promoting mechanism by rhizobacteria in more depth (Bloemberg and Lugtenberg 2001). Genomes of several PGPRs isolated from the rhizosphere of sugar-beet (Redondo-Nieto et al. 2013), coconut, cocoa, areca nut (Gupta et al. 2014), rice (Chen et al. 2015), and potato (Zhang et al. 2020), have recently been sequenced and analyzed. However, knowledge regarding the complete genome sequences of PGPRs isolated from forest tree is scant.

*Cercidiphyllum japonicum* is a deciduous tree of *Cercidiphyllum* genus in *Cercidiphyllum* family. It is a tertiary relic plant of great scientific value as it enables the origin of Tertiary flora to be studied. In addition, it has great economic importance due to the medicinal value of its fruits and leaves, and its bark being used to produce tannic extracts. *C. japonicum*, which has a straight trunk and typically colored leaves, is considered a landscape tree of high ornamental value. *C. japonicum* was widely distributed in the Northern Hemisphere. However, its area of distribution has decreased sharply since the late Quaternary glaciation. At present, it is only sporadically found in China and Japan (Crane 1984). Due to the small number of populations, *C. japonicum* was entered on the list of endangered plants in China (Fu 1992). According to the standards of the International Union for Conservation of Nature (IUCN), it is a globally recognized low-risk (LR) species. In this study, we isolated a plant growth-promoting bacterium (PGPB), *Pseudomonas putida* LWPZF, from *C. japonicum* rhizosphere, and assessed its PGP effects. The whole-genome analysis was conducted to identify genes potentially encoding plant-beneficial functions. Our results suggest that *P. putida* LWPZF is a potential candidate for use as a biofertilizer for *C. japonicum* as well as other plants.

## Materials and methods

### Phosphate-solubilizing bacteria (PSB) isolation

The rhizosphere soil of *C. japonicum* was collected from the Lishan National Nature Reserve (Shanxi, China). Ten grams of soil was added into 90 mL sterilized water and shaken for 20 min. The soil suspensions were serial diluted and plated on the NBRIP (Nautiyal 1999) agar plates. The incubation temperature was 28 °C except indicated otherwise. After incubation for 4 days. Single colonies with obvious halos were selected for phosphate solubilization measurement.

### Measurement of phosphate solubilization

To quantify the soluble phosphate produced by PSB strains, 500 μL bacterial culture (approximately 3 × 10^8^ to 5 × 10^8^ cfu/mL) was inoculated into 50 mL of NBRIP medium. After 4 days’ incubation at 180 rpm, the soluble P concentration in the culture supernatant was determined using the colorimetric molybdate blue method (Olsen and Sommers 1982).

### Strain identification

Using the methods described previously (Jin et al. 2020), the morphology, utilization of 71 carbon sources, and sensitivity to 23 chemicals of LWPZF were determined. The *16S rRNA* gene was amplified with the universal primers, 27F and 1492R under the PCR conditions described previously (Jin et al. 2020).

### Measurement of IAA production

Cultures of bacterial strains maintained overnight in nutrient broth (NB) medium were inoculated at 1:100 (v/v) into 50 mL King B medium (Glickmann and Dessaux 1995) containing 100 mg/L l-tryptophan. The bacterial cultures were incubated at 120 rpm for 7 days and centrifuged to obtain the supernatant. The IAA concentration was determined with the Salkowski colorimetric technique (Glickmann and Dessaux 1995).

### Measurement of ACCD activity

The activity of ACCD was determined by measuring the contents of α-ketobutyrate and total protein in bacterial lysates (Penrose and Glick 2003).

### Inoculation treatment

*C. japonicum* seedlings were cultivated in the nursery greenhouse of Changzhi natural resources comprehensive service center (Changzhi, Shanxi, China).

*P. putida* LWPZF was cultured in NB medium for 36 h at 180 rpm before being centrifuged (12,000×*g*, 5 min) to collect cell pellets. Cell pellets were washed thrice with sterile normal saline, and diluted to 10^8^ cfu/mL. Five milliliters of the bacterial suspension was inoculated into potted 1-year-old seedlings of *C. japonicum*. Seedlings in the control group were inoculated with an equal volume of sterile water. Ten replicates were performed for each treatment. The height and ground diameter of the seedlings were determined 30 days following inoculation of the bacterial suspensions.

### Antifungal activity of *P. putida* LWPZF on phytopathogenic fungi

The antifungal activity of LWPZF against the phytopathogenic fungi, *Fusarium oxysporum*, *Rhizoctonia solani*, *Rhizoctonia* sp., and *Pyricularia grisea*, was evaluated using Petri dishes (9 cm in diameter). Briefly, a 5 mm diameter disk of pathogenic fungi was inoculated in the center of potato dextrose agar (PDA) plates. Colonies of *P. putida* LWPZF were streaked 1 cm from the margin of the PDA plates. Control plates, incubated with each pathogenic fungus, were prepared. The plates were kept at 28 °C for incubation. When the fungal hyphae on the control plate reach the Petri dishes’ edge, the percent inhibition (PI%) was calculated: PI% = [(A − B)/A] × 100. A and B are mycelial diameters on the control and experimental plates, respectively.

### Whole-genome sequencing

Genomic DNA was isolated from the overnight LWPZF LB liquid culture using a Wizard HMW DNA extraction kit (Promega). The genome was sequenced by the PacBio RS II sequencer based on single molecule, real-time (SMRT) DNA sequencing technology. Each SMRT cell contains 150,292 SMRT zero-mode waveguides (ZMWs). The ZMWs that captured a single DNA molecule were screened for sequencing. SMRT Analysis v2.2 software was used to filter and process the raw data. The resulting sequences were de novo assembled using the hierarchical genome-assembly process (Chin et al. 2013). The LWPZF genome was annotated by the NCBI Prokaryotic Genomes Annotation Pipeline. Genes involved in PGP were identified with NCBI annotation and IMG (Integrated Microbial Genomes) website. The details for phylogenetic analysis and whole-genome sequence comparisons were described in Additional file 1.

### GenBank accession number

The GenBank accession numbers of the whole genome and 16S rRNA of *P. putida* LWPZF are CP069080 (chromosome), CP069081 (plasmid) and OK271224 (16S rRNA), respectively.

## Results

### Isolation and identification of *P. putida* LWPZF

**Fig. 1:**
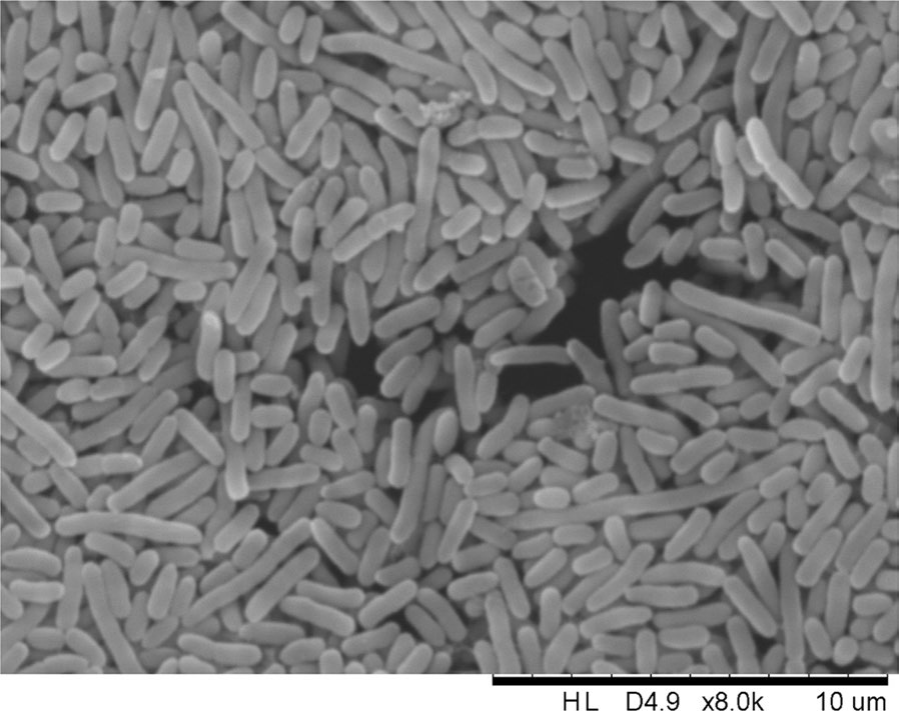
Scanning electron micrograph of *Pseudomonas putida* LWPZF cells

A total of 20 PSB were isolated from the rhizosphere of *C. japonicum*, with LWPZF exhibiting the highest phosphate solubilization capacity. A microscopic examination revealed that LWPZF is rod-shaped (Fig. 1). A 16S rRNA sequence comparison was conducted between LWPZF and strains in the EzTaxon-e database. The 16S rRNA sequence of *Pseudomonas* sp., R17(2017), had the highest similarity (99.93%) to LWPZF. In addition, LWPZF formed a subclade with its nearest neighbors, *Pseudomonas* sp. R17(2017) in the phylogenetic tree (Additional file 1: Fig. S1). In addition to l-fucose and d-fructose-6-phosphate utilization and potassium tellurite sensitivity, the use of 71 carbon sources and susceptibility to 23 compounds by strain LWPZF were highly comparable to those of *P. putida* (Additional file 1: Table S1). Based on these analyses, we concluded that LWPZF was a *P. putida* strain. LWPZF has been preserved in CCTCC with the deposit number of CCTCC M 2016008.

### Characterization for PGP and antifungal traits of LWPZF

**Fig. 2:**
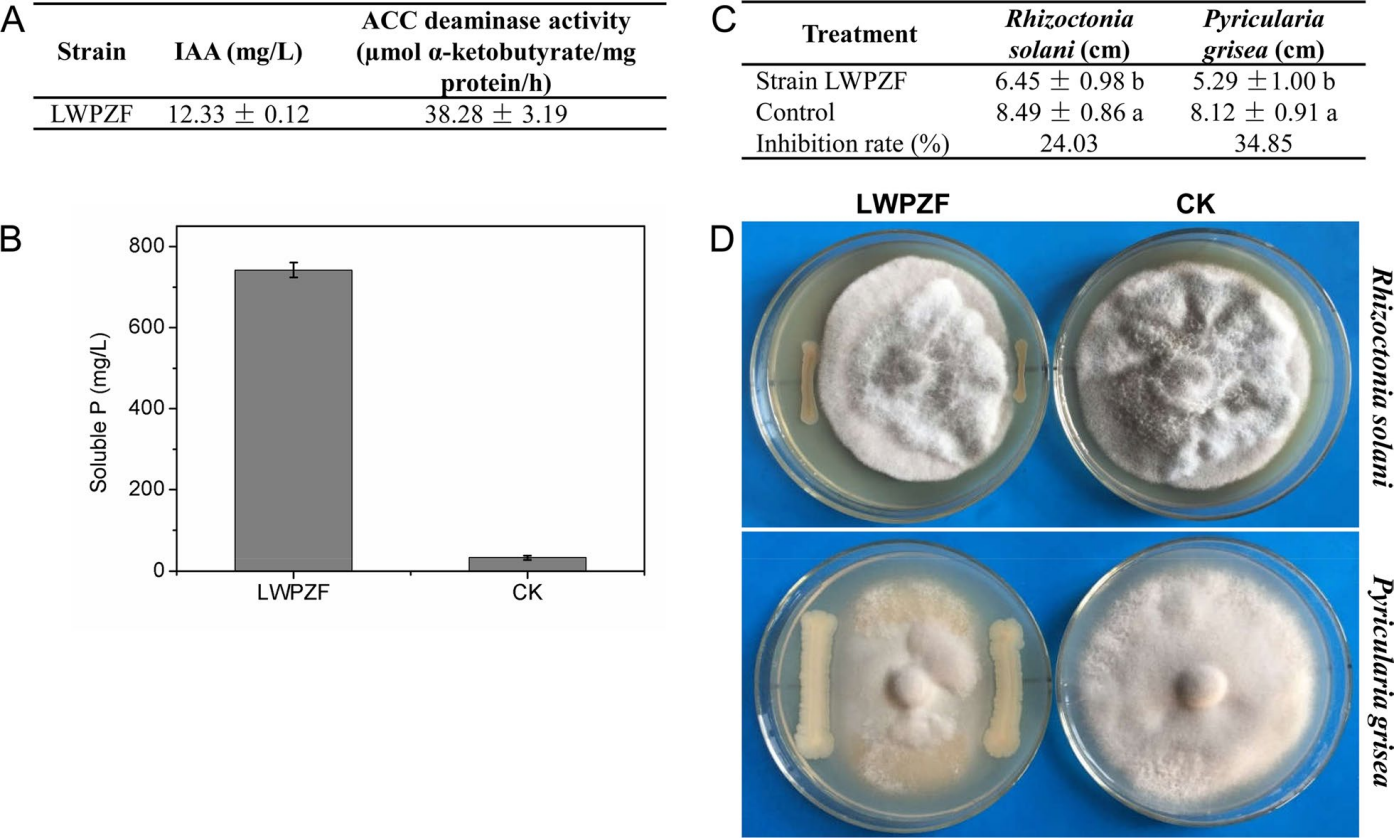
Traits involved in PGPR activity and antagonistic function of LWPZF. **A** IAA production and ACCD activity; **B** Phosphate solubilization; **C**, **D** Antagonistic function of LWPZF to *Rhizoctonia solani* and *Pyricularia grisea*. Different letters indicate significant differences (*P* < 0.05)

Some characteristics of LWPZF involved in PGP activities were analysed (Fig. 2). The concentration of IAA in the supernatant was 12.33 ± 0.12 µg/mL after 7 days of incubation in King B medium containing 100 mg/L l-tryptophan. ACCD activity of LWPZF was 38.28 ± 3.19 µmol α-ketobutyrate/mg protein/h (Fig. 2A). The soluble P content in the supernatant was 742.04 ± 18.09 mg/L after 4 days of incubation in NBRIP medium (Fig. 2B). These results suggest that *P. putida* LWPZF possesses many characteristics involved in PGP.

We also tested the antifungal activity of LWPZF against that of five phytopathogenic fungi. The growth of *R. solani* and *P. grisea* was significantly inhibited by LWPZF (Fig. 2D). The percent inhibition of *R. solani* and *P. grisea* were 24.03% and 34.85%, respectively (Fig. 2C).

### *P. putida* LWPZF increases growth of *C. japonicum* seedlings

We applied a suspension of *P. putida* LWPZF cells to *C. japonicum* seedlings and assessed their growth after 30 days. Significant increases in the heights and ground diameters of *C. japonicum* seedling were observed (Table 1). Compared with the untreated control, LWPZF inoculation led to increases of 31.30% and 43.48% in seedling height and ground diameter, respectively. In addition, we tested the growth-promoting capacity of LWPZF on the vegetable crop, cucumber, LWPZF could improve the germination of cucumber seeds (Additional file 1: Fig. S2).

**Table 1:** Effects of LWPZF inoculation on the growth of *Cercidiphyllum japonicum*

Treatment	Seedling height (cm)	Ground diameter (mm)
*P. putida* LWPZF	78.02 ± 7.06 a	4.62 ± 0.69 a
Control	59.42 ± 8.78 b	3.22 ± 0.50 b

Different letters indicate significant differences (*P* < 0.05)

### General features and functional analysis of LWPZF genome

**Fig. 3:**
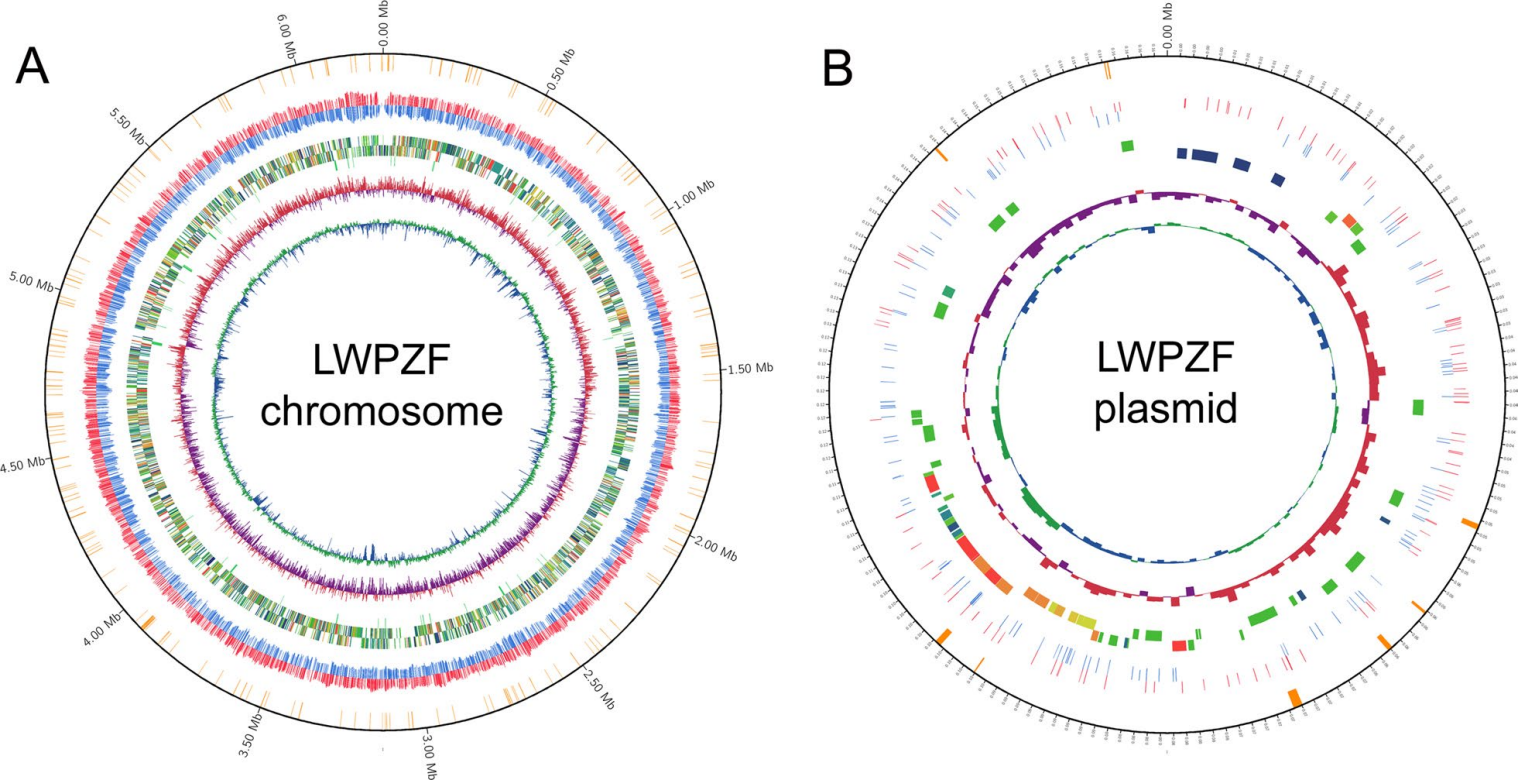
Circular genome map of *P. putida* LWPZF. **A** Genetic map of the chromosome. **B** Genetic map of the plasmid. The first circle represents distribution of genes belonging to restrictive modification system. The second and third circles depict the base modification distribution of positive and negative chains, respectively. The fourth and fifth circles show the CDS distribution of positive and negative chains, respectively. The sixth circle represents ncRNA. The seventh and eighth circles represent GC skew and GC content, respectively

The whole genome of LWPZF contained a circular chromosome and a plasmid (Fig. 3). The chromosome was 6,259,530 bp, with a GC ratio of 61.75%. The size and GC ratio of the plasmid were 160,969 bp and 58.25%, respectively. General characteristics of the complete genome are summarized (Table 2).

**Table 2:** General genome characteristics of *P. putida* LWPZF

Characteristics	Chromosome	Plasmid
Genome size (bp)	6,259,530	160,969
G+C content (%)	61.75	58.25
rRNAs	22	–
tRNAs	79	–
CDSs	5632	169
GenBank accession	CP069080	CP069081

“–”, not detected

The predicted genes of LWPZF were functional analyzed using the COG, GO, and KEGG databases. Among the 5906 genes, COG, GO, and KEGG annotations were applicable to 3453 (58.47%), 3572 (60.48%), and 3097 (52.44%) genes, respectively (Additional file 1: Table S2). The 3453 COG annotated genes were classified into 24 specific categories (Additional file 1: Fig. S3A). The most frequently represented functional clusters were “Amino acid transport and metabolism” (447, 12.95%), “transcription” (430, 12.45%), and “signal transduction mechanisms” (307, 8.89%), followed by “general function prediction only” (284, 8.22%), “energy production and conversion” (253, 7.33%), and “cell wall/membrane/envelope biogenesis” (243, 7.04%).

GO-based classification of genes revealed 2442, 1053, and 3082 genes involved in biological processes, cellular components, and molecular functions, respectively; these three categories were further classified into 13, 2, and 12 subcategories, respectively (Additional file 1: Fig. S3B). “Cellular process” (1757, 71.95%) and “metabolic process” (1308, 53.56%) in biological processes, and “catalytic activity” (1789, 58.05%) and “binding” (1432, 46.46%) in molecular functions, were the dominant categories.

Annotation via the KEGG pathway classified the annotated genes into 50 KEGG pathways. The enriched KEGG pathways were highly represented by “protein families: signaling and cellular processes” (973, 31.42%), “protein families: genetic information processing” (649, 20.96%), and “amino acid metabolism” (321, 10.36%); (Additional file 1: Fig. S3C).

### Phylogenetic analysis and genome comparisons

**Fig. 4:**
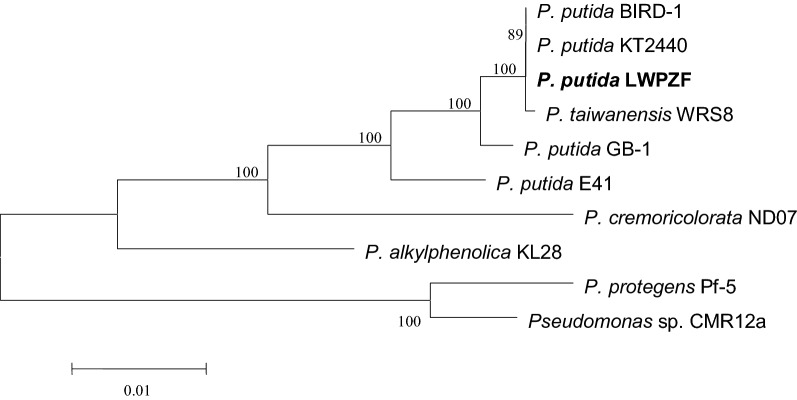
Phylogenetic relationship of marker proteins of strain LWPZF and related *Pseudomonas* species

To better define the taxonomic classification of LWPZF, we constructed a phylogenetic tree based on the amino acid sequences of three core housekeeping genes. LWPZF was clustered with *P. taiwanensis* WRS8, *P. putida* KT2440 and BIRD-1 (Fig. 4). The *P. taiwanensis* WRS8, which is a cadmium-resistance bacteria isolated from wheat rhizosphere (Cheng et al. 2021), was the closest relative. Besides, the ANI values between LWPZF and WRS8 were as high as 98.21% (ANIb) and 98.5% (ANIm) (Additional file 1: Table S3).

**Fig. 5:**
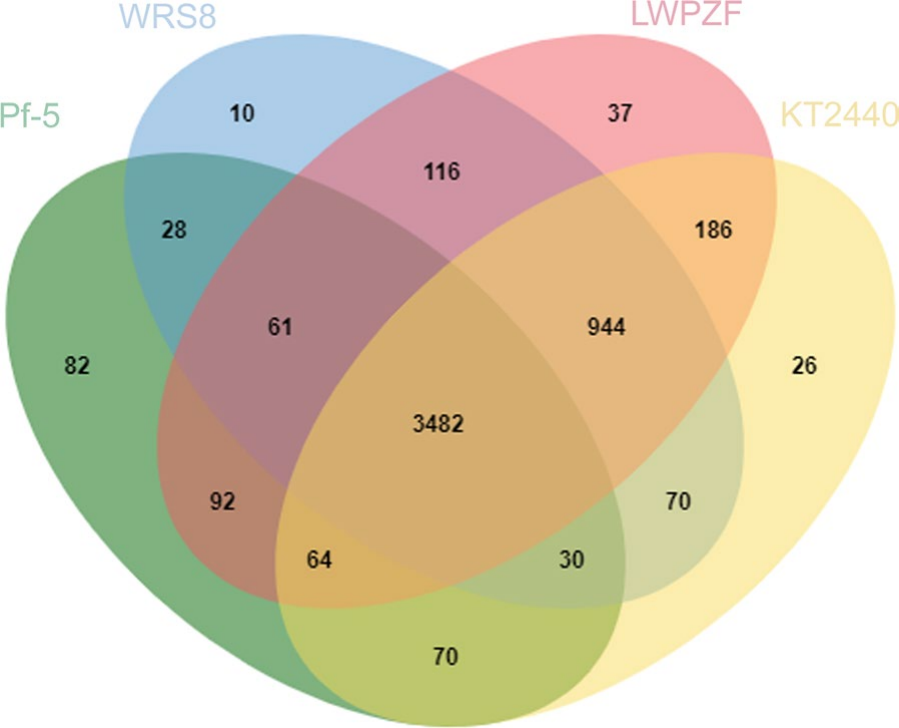
Orthologous protein clusters shared by the genome of LWPZF, KT2440, WRS8 and Pf-5

We conducted the orthologous protein clusters (OPCs) analysis between the genome of LWPZF and other three plant-associated *Pseudomonas* strains (*P. putida* KT2440, *P. taiwanensis* WRS8, and *P. protegens* Pf-5). These four strains shared 3482 OPCs in the Venn diagram (Fig. 5). LWPZF shared the most OPCs (4676) with strain KT2440. Thirty-seven OPCs were identified as being unique to LWPZF. Among these, 22 OPCs were classified as hypothetical proteins, 15 OPCs were identified as proteins involved in DNA integration, DNA recombination, DNA-mediated transposition, metal ion binding and transport, mercury ion response, secondary active sulfate and mercury ion transmembrane transporter activities, pathogenesis, polysaccharide transport, and viral genome integration into host DNA.

### Identification of genes involved in PGP

#### IAA

**Fig. 6:**
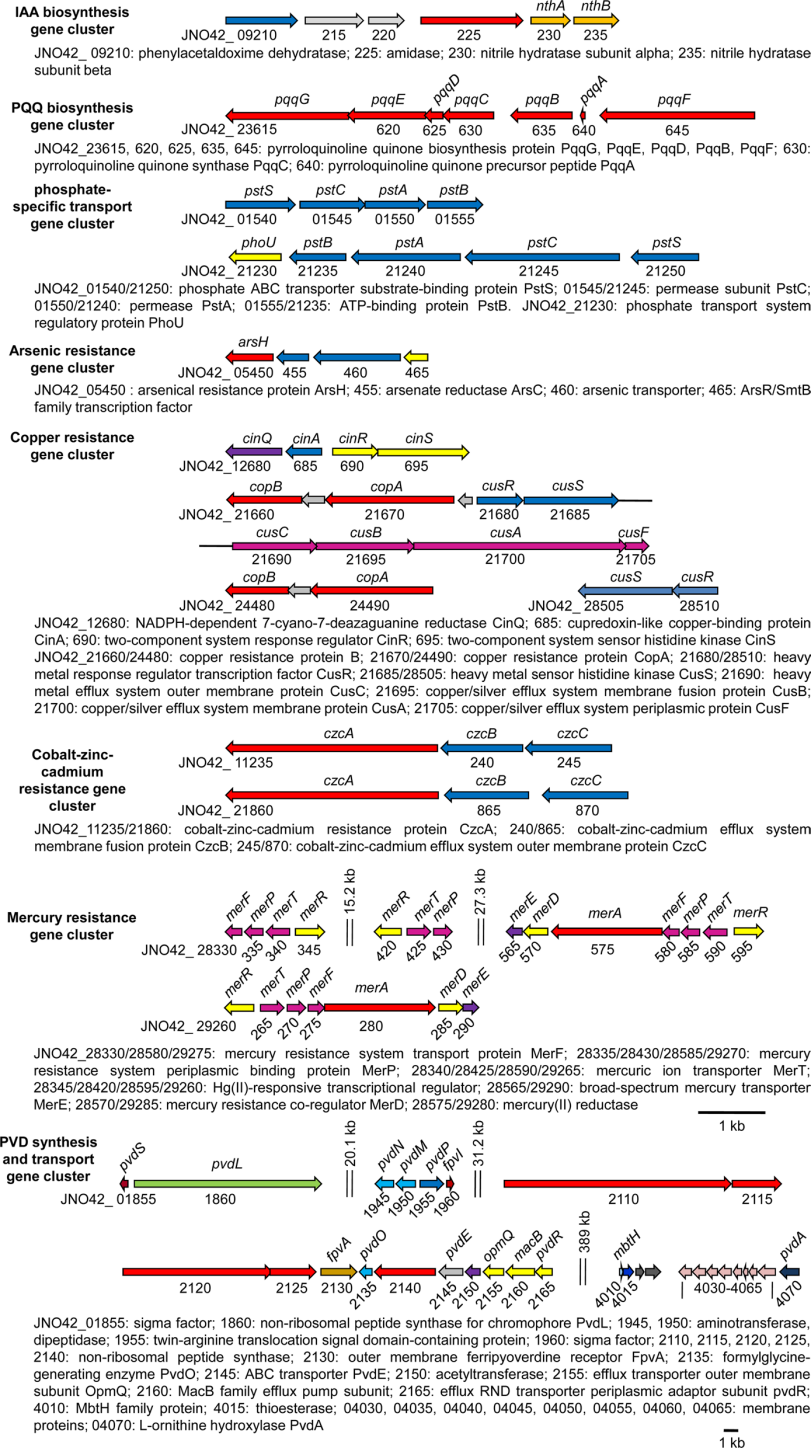
Gene clusters associated with plant growth promotion and heavy metal resistance in *P. putida* LWPZF

**Fig. 7:**
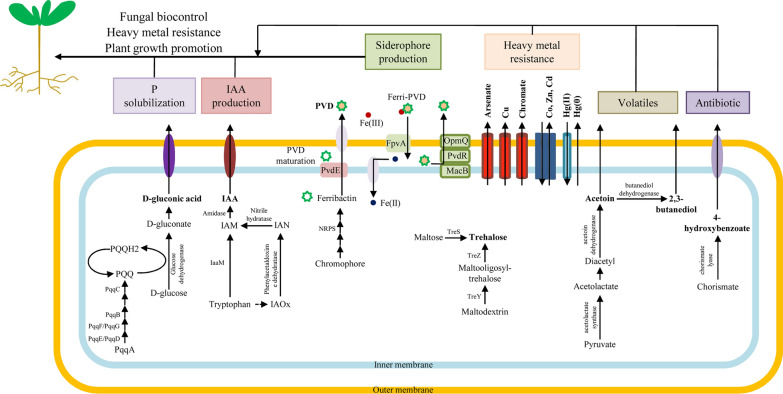
Speculated schematic summary of metabolic pathways involved in plant growth promotion, heavy metal resistance, and fungal biocontrol in *P. putida* LWPZF. The texts above or on both sides of the arrows represent enzymes catalyzing each reaction steps which were identified in the LWPZF genome. The ORF ID of these enzymes were shown in Additional file 1: Table S4. The PVD transport process was modified according to the publication of Imperi et al (2009). PQQ, pyrroloquinoline; PQQH2, pyrroloquinoline quinol; IAM: indole-3-acetamide; IaaM: tryptophan 2-monooxygenase; IAOx: indole-3-acetaldoxime; IAN: indole-3-acetonitrile; NRPS: non-ribosomal peptide synthase; PVD: pyoverdine

Genes involved in IAA biosynthesis were screened in the LWPZF genome. The indole-3-acetamide (IAM) and indole-3-acetonitrile (IAN) pathways, which may involve 5 genes, were identified in the genome of LWPZF. In the IAM pathway, *JNO42_27680* and *JNO42_09225* encode tryptophan 2-monooxygenase (IaaM) and amidase, respectively. IaaM is responsible for the converting of Trp to IAM, and amidase catalyzes the formation of IAA from IAM. The product of JNO42_09210 is predicted to be phenylacetaldoxime dehydratase catalyzing the synthesis of IAN from indole-3-acetaldoxime, which is converted from tryptophan by uncharacterized enzymes. Next, IAN is converted to IAM by nitrile hydratase (NthAB) (JNO42_09230 and JNO42_09235) (Figs. 6, 7; Additional file 1: Table S4).

#### Phosphate solubilization

Phosphorus is essential for the synthesis of nucleic acid, coenzymes, and ATP in plants. However, the majority of phosphorus in soil is unavailable for plants to uptake. Many rhizobacteria are able to convert these phosphates into the available forms (Vassilev et al. 2012). The release of gluconic acids secretion is the best-characterized mechanism by which bacteria solubilize phosphate (Alori et al. 2017). Gluconic acid is produced from glucose under the catalysis of glucose dehydrogenase which requires the cofactor pyrroloquinoline (PQQ) (An and Moe 2016). Genes encoding glucose dehydrogenase (JNO42_16315) and the *pqqFABCDEG* operon (JNO42_23645–JNO42_23615) were identified in the LWPZF genome. LWPZF genome also carries two *pstSCAB* operons (JNO42_05140–JNO42_05155 and JNO42_21250–JNO42_21235), which encode a phosphate-specific transport system (Figs. 6, 7; Additional file 1: Table S4). An additional predicted paralog of *pqqA* (JNO42_05265), referred herein as *pqqA2*, was far away from *pqqFABCDEG* operon.

The *pqqFABCDEG* arrangement in LWPZF was identical to that of *P. putida* KT2440 (An and Moe 2016). The PqqFABCDEG in LWPZF demonstrated a high degree of similarity with that of KT2440 at the amino acid level as follows: PqqF, 96.1%; PqqA, 100%; PqqB, 99.3%; PqqC, 100%; PqqD, 100%; PqqE, 96.9%; and PqqG, 92%. In addition, the amino acid sequence of the glucose dehydrogenase gene in LWPZF was 90% identical to that of *gcd* in KT2440. Thus, we deduced that LWPZF possesses a phosphate solubilization mechanism similar to that of KT2440.

#### ACC deaminase

A putative ACCD gene (*JNO42_13430*) was found in the LWPZF genome (Additional file 1: Table S4). The product of this gene belongs to pyridoxal-phosphate (PLP) dependent enzyme. A search in the KEGG database showed that the ACCD gene in LWPZF was highly homologous to that in the genome of KT2440 (PP_RS10440) and *P. putida* BIRD-1 (PPUBIRD1_3642), with amino acid sequence identities of 95.3% and 94.9%, respectively. The ACCD from LWPZF was compared to the AcdS from *Pseudomonas* sp. UW4 (Shah et al. 1998), which is closely related to LWPZF. They share only 20.4% identity.

#### Siderophore

We found that LWPZF harbored the genes associated with the synthesis of siderophore pyoverdine (PVD), which are present in four gene clusters, separated by 20.1, 31.2, and 389 kb (Figs. 6, 7; Additional file 1: Table S4). These four gene clusters included the following: (i) two genes (JNO42_01855 and 01960) coding for sigma factor PvdS (Leoni et al. 2000) and FpvI (Edgar et al. 2014; Redly and Poole 2005) which regulate PVD production and uptake; (ii) the chromophore biosynthetic gene, *pvdL* (JNO42_01860); (iii) five genes (JNO42_02110, 02115, 02120, 02125, and 02140) coding for non-ribosomal peptide synthase (NRPS) responsible for PVD biosynthesis, and (iv) the ABC inner membrane transporter gene, *pvdE* (JNO42_02145), whose product is responsible for transporting the PVD precursor into peripheral cytoplasm (Yeterian et al. 2010); (v) *pvdONMP* genes (JNO42_02135, 01945, 01950, and 01955) involved in PVD precursor maturation process (Cornelis 2013; Ringel et al. 2018); (vi) the outer membrane (OM) receptor gene *fpvA* (JNO42_02130), whose product translocates ferri-PVD into the periplasmic space (Greenwald et al. 2008; Shen et al. 2002); (vii) the genes *opmQ* (JNO42_02155), *macB* (JNO42_02160), and *pvdR* (JNO42_02165), whose products constitute a three-component transport pump that transports PVD from the peripheral cytoplasm to the extracellular milieu (Imperi et al. 2009); (viii) *mbtH* (JNO42_04010) associated with NRPSs of cytoplasmic ferribactin synthesis (Felnagle et al. 2010); (ix) One thioesterase gene (JNO42_04015) that may participate in the manufacture of the initial dipeptide during the chromophore’s nonribosomal construction (Ravel and Cornelis 2003); (x) eight membrane protein genes (JNO42_04030–JNO42_04065) which might be regulated by PvsS (Ochsner et al. 2002); and (xi) *pvdA* gene (JNO42_04070) coding for the l-ornithine *N*^*5*^-oxygenase involved in pyoverdine biosynthesis (Visca et al. 1994). A gene encoding an acetyltransferase (JNO42_02150) was present in the genome of LWPZF. This protein showed 41% and 38.5% identity with PvdYII from *Pseudomonas* sp. UW4 (Duan et al. 2013) and *Pseudomonas aeruginosa* Pa4 (Lamont et al. 2006), respectively. These results indicate that LWPZF probably produce type II PVD.

The PVD genomic regions in four pseudomonads, *P. aeruginosa* PAO1, *P. fluorescens* Pf0–1, *P. putida* KT2440, and *P. syringae* DC3000, have been analysed and compared previously (Ravel and Cornelis 2003). The general arrangement of genes responsible for PVD production in LWPZF differs from that found in these four pseudomonads. The PVD synthesis genes are localized in one large cluster in *P. syringae* DC3000, two clusters in *P. aeruginosa* PAO1, and three in *P. fluorescens* Pf0–1 and *P. putida* KT2440. However, PVD genes are distributed in four locations in the LWPZF genome. In addition, the genes *pvdONMP*, *ompQ*, *macB*, *pvdR*, and *fpvI* are located in the same gene cluster in PAO1, as well as in the other pseudomonads (Ravel and Cornelis 2003), whereas *pvdNMP* and *fpvI* are located in a separate cluster in LWPZF (Fig. 6).

A comparison between PVD genes in LWPZF and *P. aeruginosa* PAO1, revealed that 24 of the 29 putative PVD genes in LWPZF were related to those in PAO1. Translated amino acid sequence identity levels between the corresponding genes ranged from 34.7% (*LWPZF JNO42_02130* to *PAO1 PA2398*) to 85.4% (*LWPZF JNO42_04035* to *PAO1 PA2409*). Two opening reading frames (ORFs) of *JNO42_04020* and *JNO42_04025*, upstream of the membrane protein gene cluster *JNO42_04030*–*JNO42_04065* were unique and had no homologs among the PVD genes in PAO1. *JNO42_04020*, encodes an isochorismatase family protein. The putative protein encoded by *JNO42_04025* showed no homology in the sequence database. However, the products of these two genes were highly similar to those of the PVD genes, *PP_RS19810* and *PP_RS19805*, in *P. putida* KT2440. Among the six genes encoding NRPS in LWPZF, only the chromophore precursor synthetase gene, *pvdL* (JNO42_01860) were highly similar (70.7%) to the corresponding gene in PAO1 (PA2402) at the amino acid sequence level. The other genes encoding NRPSs showed a low degree of identity. These results are in line with the findings of Ravel and Cornelis (2003) indicating that PvdL was highly conserved, whereas the other NRPSs showed low degrees of similarity.

#### Trehalose

Five different routes of trehalose biosynthesis have been described in bacteria (Paul et al. 2008). Genes encoding two trehalose biosynthetic routes were present in LWPZF genome: TreS and TreY–TreZ (Fig. 7; Additional file 1: Table S4). Trehalose synthase (TreS), the coding product of JNO42_02760, catalyzes the formation of trehalose from maltose. In the TreY–TreZ route, malto-oligosyltrehalose synthase (TreY) (JNO42_02790) catalyzes the conversion of maltodextrin to maltooligosyl-trehalose, followed by the malto-oligosyltrehalose trehalohydrolase (TreZ) (JNO42_02800) which hydrolyzes maltooligosyl-trehalose to form trehalose.

#### Acetoin and 2,3-butanediol

Genes coding for acetoin biosynthesis, including acetolactate synthase (pyruvate to acetolactate) (JNO42_07075, 17655, 17660, and 27675) and acetoin dehydrogenase (diacetyl to acetoin) (JNO42_24570) have been identified in the genome of LWPZF (Fig. 7; Additional file 1: Table S4). An analysis of deduced protein domains revealed that the product of JNO42_24570 is associated with the activities of both acetoin dehydrogenase and butanediol dehydrogenase, which function as butanediol dehydrogenase to convert acetoin to 2,3-butanediol.

### Identification of genes responsible for tolerance against metal toxicity

We found several genes associated with tolerance to HMs in the LWPZF genome (Fig. 6; Additional file 1: Table S4). Four genes coding for arsenical resistance were discovered, including genes encoding arsenical resistance protein, ArsH (JNO42_05450), arsenate reductase (JNO42_05455), arsenic transporter (JNO42_05460), and ArsR transcription factor (JNO42_05465).

Sixteen genes were found as potentially relevant in copper resistance, which are spread throughout four areas of the genome (Fig. 6; Additional file 1: Table S4). The first region contains four genes: *cinQ*, *A*, *R*, and *S* (JNO42_12680–JNO42_12695). *CinQ* encodes an NADPH-dependent 7-cyano-7-deazaguanine reductase. The product of *cinA* is a cupredoxin-like copper-binding protein. The genes *cinR* and *cinS*, which encode the two-component system CinR/S are associated with copper tolerance. In the second region, *JNO42_21660* and *21670* encoded copper resistance protein B involved in copper binding and multicopper oxidase protein CopA, respectively. *JNO42_21680/21685* coded for the HM response regulator transcription factor/sensor histidine kinase system CusR/S. The products of JNO42_21690–JNO42_21705 were predicted to constitute the copper/silver efflux system CusCBAF (Franke et al. 2003). Additional *copBA* and *cusR/S* genes were identified to be located in the third and the fourth regions respectively.

The gene encoding the chromate resistance protein, ChrB (JNO42_28490), was also identified (Additional file 1: Table S4). The *chrB* gene encodes a chromate-sensing regulator (Branco and Morais 2013), the protein sequence of *chrB* in LWPZF showed 93.6% identity with the *chrB* gene (F753_14730) in strain *P. chloritidismutans* AW-1. Two chromate efflux transporter ChrA genes (JNO42_10645 and 28,495) found in the genome of LWPZF, showed 76.9% and 75.3% identity with *chrA* (PputUW4_03067) of *Pseudomonas* sp. UW4 at the amino acid level.

Two gene loci encoding the cobalt-zinc-cadmium efflux pump CzcCBA (JNO42_11245–JNO42_11235 and JNO42_21870–JNO42_21860) were detected (Fig. 6). The gene, *czcC* (JNO42_11245 and 21870), *czcB* (JNO42_11240 and 21865), and *czcA* (JNO42_11235 and 21860), encode the outer membrane protein, membrane fusion protein, and transporter of the cobalt-zinc-cadmium efflux system, respectively. The product of JNO42_21780 is presumed to be the cation transporter CzcD, which also regulates the expression of the CzcCBA system (Anton et al. 1999).

Four gene clusters involved in mercury resistance systems were detected in the LWPZF genome. The first three gene clusters are located on the chromosome, while the fourth is located on the plasmid. These four gene clusters have nearly identical gene compositions. *MerR*, *T*, *P*, *F*, *A*, *D*, and *E* are among the seven genes in the third and fourth gene clusters (JNO42_28595–JNO42_28565 and JNO42_29260–JNO42_29290). There are only four (*merRTPF*; JNO42_28345–JNO42_28330) and three genes (*merRTP*; JNO42_28420–JNO42_28430) in the first and second gene clusters, respectively (Fig. 6; Additional file 1: Table S4). *MerP* encodes periplasmic Hg(II)-binding protein. *MerT*, *F*, and *E* code for inner membrane proteins, which are responsible for the transport of Hg(II) from extracellular to the cytoplasmic side. Hg(II) in the cytoplasm is reduced to volatile, relatively inert Hg(0) by Hg(II) reductase encoded by *merA*, and then diffuses across the cell membrane to the outside of the cell. *MerR* encodes a Hg(II)-responsive transcriptional regulator controlling the expression of *mer* operon. The product of *merD* is a transcriptional regulator that functions antagonistically with MerR. It was speculated that the antagonistic effect of MerD on MerR promoted the inhibition of MerA activity immediately following the reduction of Hg(II), preventing MerA from exerting its oxidase activity and producing toxic peroxides (Barkay et al. 2003). Significantly, the coding proteins of these mercury resistance genes identified in the LWPZF genome belonged to the unique OPCs of LWPZF when OPCs were compared between the genome of LWPZF and other three plant-associated *Pseudomonas* strains (Fig. 5), indicating the broad-spectrum resistance potential of LWPZF to a variety of heavy metals.

### Identification of genes responsible for fungal biocontrol

Many PGPRs can synthesize antibiotics to hinder the development of plant pathogens. Genes coding for phenazine and 4-hydroxybenzoate biosynthesis were discovered in LWPZF genome. *P. aeruginosa* PAO1 contains *phzABCDEFG* operon genes involved in phenazine biosynthesis (Mavrodi et al. 2001). We only found *phzF* (JNO42_01500) in the genome of LWPZF (Additional file 1: Table S4). The *ubiC* gene (JNO42_21190), coding for chorismate lyase which catalyzes chorismate to 4-hydroxybenzoate, was identified in LWPZF genome (Fig. 7; Additional file 1: Table S4). A search for *ubiC* in the *Pseudomonas* genome revealed that it was present in many plant-associated *Pseudomonas* species, including *Pseudomonas* sp. UW4 (PputUW4_05351), *P. putida* KT2440 (PP_RS27695), and *P. protegens* Pf-5 (PFL_RS30945), indicating the prevalence of 4-hydroxybenzoate synthesis in plant-associated Pseudomonads.

## Discussion

In this study we reported the isolation, identification, characterization of PGP traits, whole genome sequencing and analysis of a plant growth-promoting bacterium *P. putida* LWPZF from *Cercidiphyllum japonicum* rhizosphere. The PGP traits characterization and whole genome analysis reported here of LWPZF, to our knowledge is the first for rhizobacteria isolated from the endangered plant *C. japonicum*. Strain LWPZF possesses several PGP characteristics, including IAA production, P solubilization, production of ACCD. Inoculation assay revealed that strain LWPZF could promote the growth of *C. japonicum* significantly. Besides, we tested the growth-promoting capacity of strain LWPZF on the vegetable crop cucumber (Additional file 1: Fig. S2), LWPZF could improve the germination of cucumber seeds. Overall, *P. putida* LWPZF shows great potential as an application in the development of biofertilizers, not only in the case of the endangered plant, *C. japonicum*, but also in other vegetable crops, such as cucumber.

IAA secretion is one of the important PGP properties for certain PGPRs (Kochar et al. 2011). Tryptophan (Trp) is used as the general precursor for IAA synthesis by the majority of bacteria. To date, based on difference in intermediates, there are five proposed Trp-dependent IAA biosynthesis pathways in bacteria, including IAM, IAN, indole-3-pyruvic acid (IPyA), tryptamine (TAM), and tryptophan side chain oxidase (TSO) pathways (Spaepen et al. 2007). In *Azospirillum brasilense* and *Micrococcus aloeverae* DCB-20, a Trp-independent pathway was discovered, however, the enzyme catalyzing this pathway has not been identified (Ahmad et al. 2020; Prinsen et al. 1993). Two Trp-dependent IAA biosynthesis pathways, IAM and IAN, were identified in the LWPZF genome. The IAM pathway is a well-studied pathway among the bacterial IAA synthesis pathways, in which IAM is usually catalyzed by IAM hydrolase (IaaH) to form IAA. However, *iaaH* was not detected in the LWPZF genome. The IAN pathway was also discovered in *Pseudomonas* sp. strain UW4 (Duca et al. 2014) and *Variovorax boronicumulans* CGMCC 4969 (Sun et al. 2018). IAN can not only form IAA via the nitrile hydratase/amidase pathway, but also directly produce IAA via nitrilase catalysis in these two strains. However, genes encoding nitrilase were not detected in the LWPZF genome. The IAA synthesis pathway seemed to integrate the IAM and IAN pathways in LWPZF. IAM was synthesized by IAA or IAN, and further converted to IAA via amidase catalyzing, indicating the diversity and flexibility of IAA synthesis in bacteria.

ACCD is able to break down ACC, an ethylene precursor, to reduce ethylene levels and contributes to plant growth (Glick 2014). It is widely assumed that ACCD is encoded by *acdS* (Singh et al. 2015). The ACCD coding gene in LWPZF only shares a low identity with *acdS* in the *Pseudomonas* sp. UW4. AcdS also belongs to PLP dependent enzyme family. It was pointed out that five amino acid residues, K51, S78, Y294, E295, and L322, (numbered in UW4) in the AcdS sequence were essential for ACCD activity (Nascimento et al. 2014). The sequence alignment of ACCD from LWPZF and AcdS in UW4 revealed that ACCD from LWPZF contained three key amino acid residues K51, S78, and Y294 but lacked E295 and L322. Todorovic and Glick (2008) concluded that a true ACCD should contain E295 and L322. The mutation E295S/L322T in UW4 AcdS resulted in the loss of ACCD activity. Whether the ACCD activity detected in LWPZF (Fig. 2A) is encoded by the putative ACCD gene (*JNO42_13430*) remains to be further investigated.

Siderophores are a type of highly specific Fe iron chelator secreted by bacteria. Many PGPRs can restrict pathogen growth by secreting siderophores to competitively capture Fe irons, making them useful in biological control (Crichton and Charloteaux-Wauters 1987). The yellow-green fluorescent PVD is the principal siderophore produced by *Pseudomonas*, which is also the most studied siderophore (Cornelis 2010; Meyer 2000; Schalk and Guillon 2013). Doubtlessly, PVD synthesis genes were identified in LWPZF genome. A comparison between PVD genes in LWPZF and *P. aeruginosa* PAO1, revealed that NRPSs except PvdL showed low degrees of similarity. PvdL and other NRPSs cooperate to complete the synthesis of the PVD peptide backbone. The low similarity of these NRPSs indicates that LWPZF may produce a different PVD peptide moiety structure.

In addition to the PGP characteristics demonstrated by experiments (Fig. 2). Genome analysis of LWPZF revealed several other PGP properties, including the synthesis of trehalose, acetoin, 2,3-butanediol, as well as antibiotics phenazine and 4-hydroxybenzoate. Trehalose is an effective osmoprotectant which can assist plants withstand a range of abiotic stressors (Iturriaga et al. 2009). Many PGPRs reduce stress and enhance plant growth in drought and high salt situations by generating trehalose (Vilchez et al. 2016). The lack of trehalose synthesis pathway in *Sinorhizobium meliloti* affected its nodulation in Alfalfa roots. Rodríguez-Salazar et al. (2009) found that the leaf and root biomass of maize plants inoculated with *Azospirillum brasilence* overexpressing the trehalose synthesis gene was significantly improved. In another study, a trehalose-overexpressing strain of *Pseudomonas* sp., UW4, significantly increased the root length and biomass of tomato plants under salt stress (Orozco-Mosqueda et al. 2019). A number of PGPRs are able to release volatiles, which regulate plant growth and physiological metabolism (Sharifi and Ryu 2018). For example, acetoin and 2,3-butanediol have been found to boost *Arabidopsis* growth (Ryu et al. 2003). Duan et al., searched for orthologs in the genome of 21 *Pseudomonas* species (Duan et al. 2013) and found that all 21 species shared the similar trehalose, acetoin, and 4-hydroxybenzoate synthetic pathways as LWPZF, indicating the ubiquity and relevance of the biosynthesis of these compounds.

With the frequent increase of industrial activities, HM pollution in soil environment is becoming more and more serious. In addition, excessive HMs can cause plant growth retardation (Rajkumar et al. 2006; Wani and Khan 2010). Many PGPRs can minimize the impacts of HMs by reducing, oxidizing, methylating, and converting to less toxic forms (Ahemad 2019). These HM-resistant PGPRs can dramatically increase plant biomass in HM polluted environments (Adhikary et al. 2019; Manzoor et al. 2019; Ren et al. 2019), and some PGPRs can even improve plant photosynthetic rate (Mesa-Marin et al. 2020). However, HM-resistant PGPRs affect the ability of plants to absorb HMs in two distinct ways. The inoculation of some metal-resistant PGPRs is able to improve plant absorption capacity to HMs, resulting an increase in HM contents in plant tissues. These PGPRs can be used as assistants to lower HM concentrations in the surrounding environment, therefore playing essential roles in ecological restoration (Harindintwali et al. 2020; Mesa-Marin et al. 2020; Rajkumar and Freitas 2008). In contrast, certain PGPRs can reduce HM uptake by plants (Manzoor et al. 2019). For example, *P. taiwanensis* WRS8 reduces chromium uptake by wheat roots and aboveground tissues by inhibiting the expression of genes related to chromium enrichment and transport in wheat (Cheng et al. 2021). This type of PGPRs are appropriate for use as a bioinoculant or biofertilizer in crops grown in HM-polluted environments. The effects of LWPZF on plant HM uptake needs to be further studied.

Although the genome of many Pseudomonads with PGP properties have been sequenced, the PGP characteristics of these stains are diverse. For instance, P solubilization characteristic was not reported in *Pseudomonas* sp. UW4. Besides, the synthesis mechanisms of IAA, ACCD, and PVD in LWPZF are different from other Pseudomonads in some extent. Aside from multiple PGP properties, genome analysis revealed that LWPZF contained a number of HM resistance genes, implying that this strain also possessed HM resistance characteristics. The growth promoting effects on *C. japonicum* of LWPZF should be the results of multiple PGP characteristics working together. We believe that LWPZF has significant potential for use in the field of microbial fertilizer. Genomic analysis in this study provide an important basis for us to study the PGP mechanisms of LWPZF in the future.

## Supplementary Information

**Additional file 1:** **Figure S1**. Neighbor-joining phylogenetic tree based on 16S rRNA gene sequences of strain LWPZF and related taxa. Bootstrap values (1000 replications) are shown as percentages at each node only if they are 50% or greater. Bar, 0.05 substitutions per nucleotide position. **Figure S2**. Effect of LWPZF on cucumber germination. **Figure S3**. COG (A), GO (B) and KEGG (C) classification of genes. ^1^biological process involved in interspecies interaction between organisms. ^2^biological process involved in intraspecies interaction between organisms. **Table S1**. Characteristics of strain LWPZF. *+: Positive; −: negative; w: weakly positive. **Table S2**. Summary of functional annotations. **Table S3**. Comparisons between the ANIs (ANIb and ANIm) of LWPZF and other related *Pseudomonas*. ^a^ANI-blast, ^b^ANI-MUMmer. **Table S4**. Genes involved in plant growth promotion, heavy metal resistance, and biocontrol in the *P. putida* LWPZF genome. **Table S5**. Accession numbers of housekeeping genes used for phylogenetic tree construction.

## Data Availability

All data supporting this study are included in the manuscript.
