# Supplementary material for: Plant growth-promoting effect and genomic analysis of the P. putida LWPZF isolated from C. japonicum rhizosphere
Source: AMB Express. 2022 Aug 2;12:101. doi: 10.1186/s13568-022-01445-3 (PMC9346032; doi:10.1186/s13568-022-01445-3)
Supplement: Supplementary file 1 — Additional file 1: Figure S1. Neighbor-joining phylogenetic tree based on 16S rRNA gene sequences of strain LWPZF and related taxa. Bootstrap values (1000 replications) are shown as percentages at each node only if they are 50% or greater. Bar, 0.05 substitutions per nucleotide position. Figure S2. Effect of LWPZF on cucumber germination. Figure S3. COG (A), GO (B) and KEGG (C) classification of genes. 1biological process involved in interspecies interaction between organisms. 2biological process involved in intraspecies interaction between organisms. Table S1. Characteristics of strain LWPZF. *+: Positive; −: negative; w: weakly positive. Table S2. Summary of functional annotations. Table S3. Comparisons between the ANIs (ANIb and ANIm) of LWPZF and other related Pseudomonas. aANI-blast, bANI-MUMmer. Table S4. Genes involved in plant growth promotion, heavy metal resistance, and biocontrol in the P. putida LWPZF genome. Table S5. Accession numbers of housekeeping genes used for phylogenetic tree construction. [file 13568_2022_1445_MOESM1_ESM.docx]

*Additional file 1.*

AMB Express

**Plant growth-promoting effect and genomic analysis of the *P. putida* LWPZF isolated from *C. japonicum* rhizosphere**

Tingting Jin ^†^, Jiahong Ren ^†^*,Yunling Li, Bianxia Bai, Ruixiang Liu, Ying Wang

Department of Life Sciences, Changzhi University, Changzhi 046011, PR China

*Correspondence: renjiahong76@hotmail.com; Tel.: 86-0355-2178013

† Contributed equally to the article

**Materials and Methods**

**Phylogenetic analysis and whole-genome sequence comparisons**

The phylogenetic tree of LWPZF and other *Pseudomonas* strains was constructed according to the amino acid sequences of three housekeeping genes, *gyrB*, *rpoB*, and *rpoD* (Table S5). The housekeeping gene sequences were concatenated in the same order and then aligned using Clustal W. A maximum-likelihood tree was generated with MEGA 6.0, using 1000 bootstrap replicates.

The average nucleotide identity (ANI) values between LWPZF genome and other related *Pseudomonas* spp. were calculated using the JSpecies Web Service ([Richter et al. 2016](#_ENREF_56)). The OrthoVenn web server Orthologous clusters analysis was performed by the OrthoVenn2 web server (e-value cut-off: 1e-5; inflation value: 1.5) ([Xu et al. 2019](#_ENREF_72)).

Reference

Xu L et al. (2019) OrthoVenn2: a web server for whole-genome comparison and annotation of orthologous clusters across multiple species. Nuclc Acids Res 47:W52-W58. https://doi.org/10.1093/nar/gkz333

*Pseudomonas* sp. Leaf58 (LMLL01000001)

*Pseudomonas putida* GB-1 (CP000926)

*Pseudomonas putida* E41 (CP024085)

*Pseudomonas capeferrum* WCS358(T) (JMIT01000002)

*Pseudomonas* sp. 02C 26 (CP025262)

*Pseudomonas reidholzensis* CCOS 865(T) (LT009707)

*Pseudomonas cremoricolorata* ND07 (CP009455)

*Pseudomonas cremoricolorata* IAM 1541(T) AB060137

*Pseudomonas sichuanensis* WCHPs060039(T) (QKVM01000121)

*Pseudomonas japonica* NBRC 103040(T) BBIR01000146

*Pseudomonas* sp. CMR12a (CP027706)

*Pseudomonas aestus* CMAA 1215(T) (KX347965)

*Pseudomonas fulva* NBRC 16637(T) (BBIQ01000036)

*Pseudomonas monteilii* USDA-ARS-USMARC-56711 (CP013997)

*Pseudomonas parafulva* NBRC 16636 (T) (BBIU01000051)

*Pseudomonas* sp. ICMP 17674 (BATF01000024)

*Pseudomonas putida* ATH-43 (LBME01000002)

*Pseudomonas putida* H8234 (NC_021491)

*Pseudomonas putida* NBRC 14164(T) (AP013070)

*Pseudomonas hutmensis* xwS2(T) QJRG01000049

*Pseudomonas alkylphenolica* KL28(T) (CP009048)

**LWPZF**

*Pseudomonas* sp. R17(2017) (NEIG01000032)

*Pseudomonas putida* KT2440 (AE015451)

*Pseudomonas putida* W5 (CP026115)

*Pseudomonas alloputida* Kh7(T) (LT718459)

*Pseudomonas hunanensis* LV(T) (JX545210)

*Pseudomonas juntendi* BML3(T) (MK680061)

*Pseudomonas plecoglossicida* NBRC 103162(T) (BBIV01000080)

*Pseudomonas monteilii* NBRC 103158(T) (BBIS01000088)

*Pseudomonas* sp. GM84 (AKJC01000277)

*Pseudomonas* sp. LB-090624 (QJRL01000068)

*Pseudomonas asiatica* RYU5(T) (MH517510)

*Pseudomonas taiwanensis* BCRC 17751(T) (EU103629)

*Pseudomonas shirazica* VM14(T) (OLKI01000048)

*Pseudomonas guariconensis* LMG 27394(T) (FMYX01000029)

*Pseudomonas putida* IEC33019 (CP016634)

*Pseudomonas entomophila* L48(T) (CT573326)

*Pseudomonas mosselii* CIP 105259(T) (AF072688)

*Pseudomonas soli* F-279208(T) (HF930598)

*Pseudomonas flavescens* LMG 18387(T) FNDG01000047

100

95

100

99

56

99

55

65

76

72

51

57

52

0.002

**Fig. S1 Neighbor-joining phylogenetic tree based on 16S rRNA gene sequences of strain LWPZF and related taxa.** Bootstrap values (1000 replications) are shown as percentages at each node only if they are 50% or greater. Bar, 0.05 substitutions per nucleotide position.


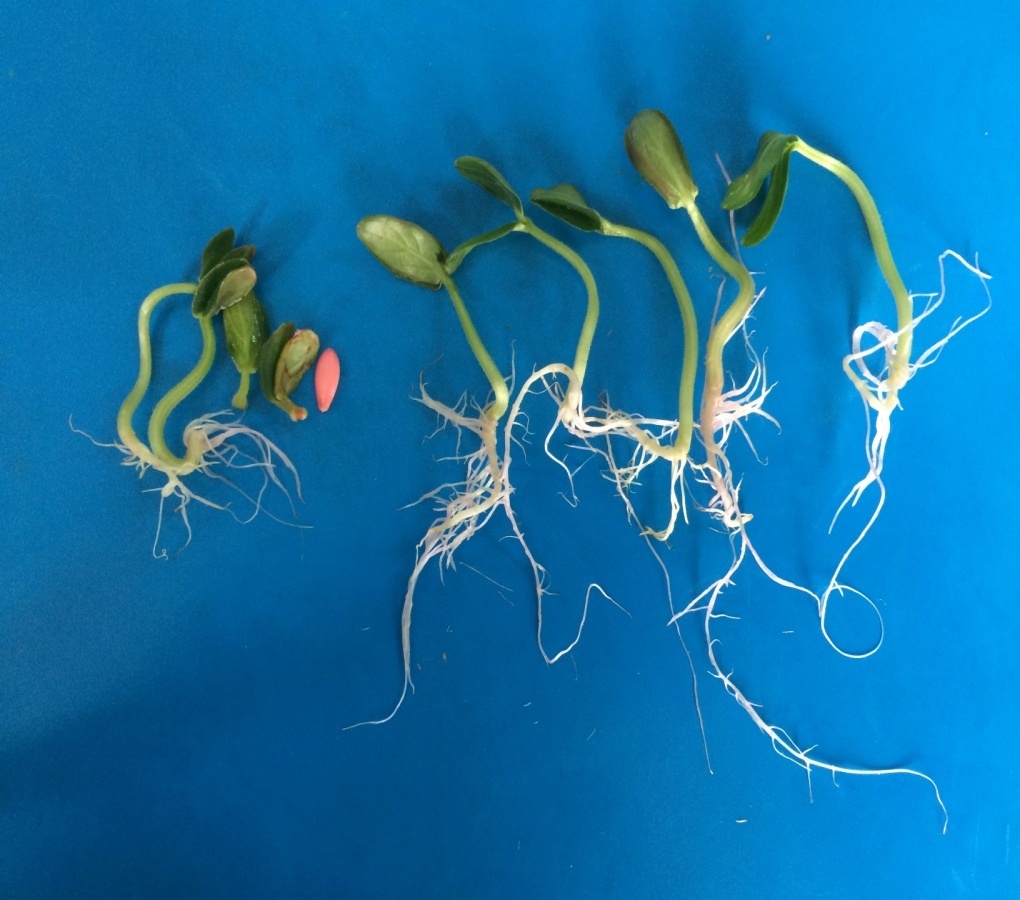


CK

LWPZF

**Fig. S2** **Effect of LWPZF on cucumber germination**

**
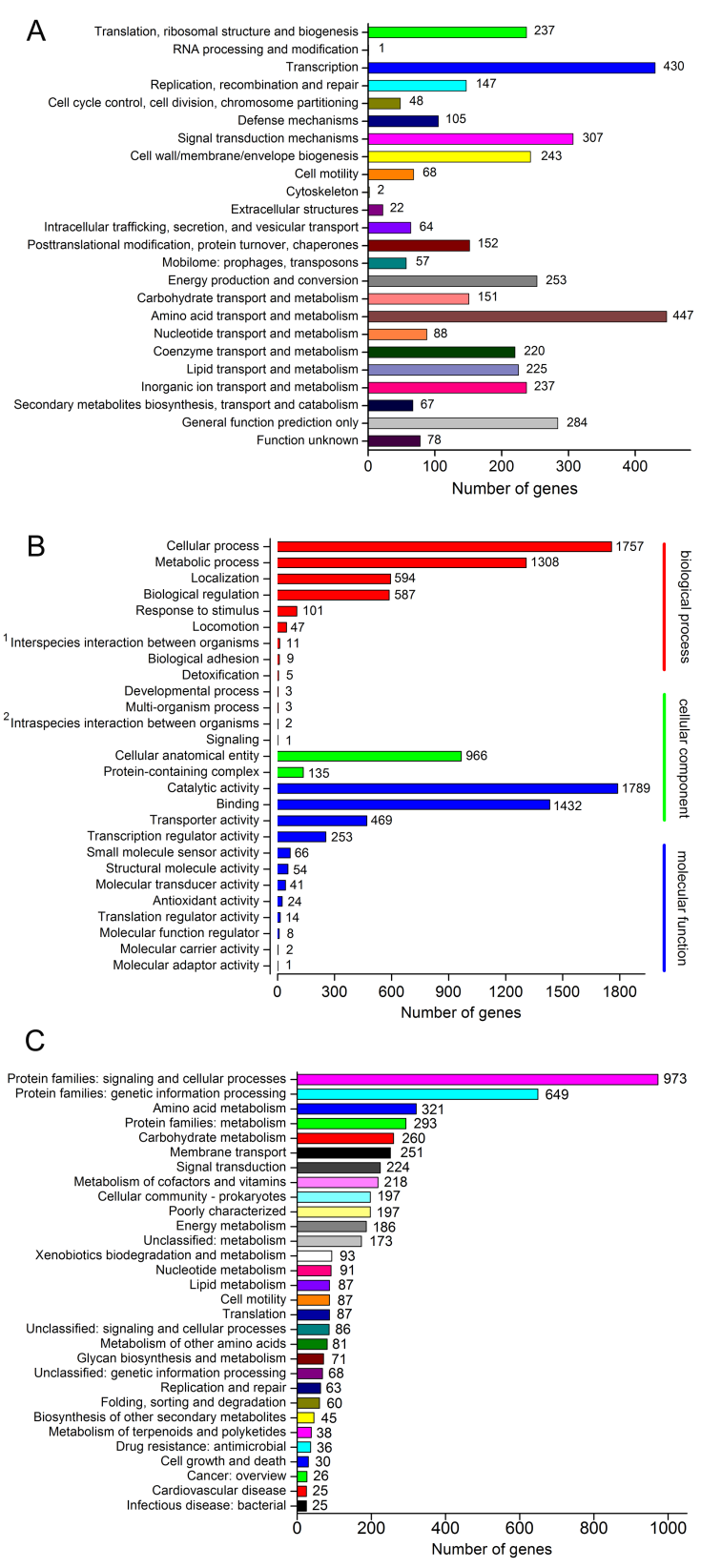
**

**Fig. S3 COG (A), GO (B) and KEGG (C) classification of genes.** ^1^ biological process involved in interspecies interaction between organisms. ^2^ biological process involved in intraspecies interaction between organisms.

**Table S1 Characteristics of strain LWPZF**

| Characteristic | Result* | Characteristic | Result | Characteristic | Result |
| --- | --- | --- | --- | --- | --- |
| Gram's dye | − | L-Fucose | w | Citric Acid | + |
| Methyl red | − | L-Rhamnose | w | α-Keto-Glutaric Acid | w |
| V-P | − | Inosine | w | D-Malic Acid | − |
| Indole | − | D-Sorbitol | − | L-Malic Acid | + |
| Nitrate reduction | + | D-Mannitol | − | Bromo-Succinic Acid | w |
| KOH | + | D-Arabitol | − | Tween 40 | w |
| Starch hydrolysis | − | myo-Inositol | − | γ-Amino-Butryric Acid | + |
| Catalase | + | Glycerol | w | α-HydroxyButyric Acid | − |
| Oxidase | + | D-Glucose-6- Phosphate | − | β-Hydroxy-D, L-Butyric Acid | w |
| *Utilization of carbon sources (BIOLOG Gen III)* | | D-Fructose-6-Phosphate | w | α-Keto-Butyric Acid | − |
|  |  | D-Aspartic Acid | − | Acetoacetic Acid | − |
| Dextrin | w | D-Serine | + | Propionic Acid | + |
| D-Maltose | − | Gelatin | − | Acetic Acid | + |
| D-Trehalose | − | Glycyl-L-Proline | − | Formic Acid | − |
| D-Cellobiose | − | L-Alanine | + | *Growth at (BIOLOG Gen III)* | |
| Gentiobiose | w | L-Arginine | + |  |  |
| Sucrose | − | L-Aspartic Acid | + | 1% NaCl | + |
| D-Turanose | − | L-Glutamic Acid | + | 4% NaCl | w |
| Stachyose | − | L-Histidine | + | 1% Sodium Lactate | + |
| D-Raffinose | − | L-Pyroglutamic Acid | + | Troleandomycin | + |
| α-D-Lactose | − | L-Serine | w | Rifamycin SV | + |
| D-Melibiose | − | Pectin | − | Minocycline | w |
| β-Methyl-D-Glucoside | − | D-Galacturonic Acid | + | Lincomycin | + |
| D-Salicin | − | L-Galactonic Acid Lactone | w | Guanidine Hydrochloride | + |
| N-Acetyl-D-Glucosamine | − | D-Gluconic Acid | + | Niaproof 4 | + |
| N-Acetyl-β-D-Mannosamine | − | D-Glucuronic Acid | + | Vancomycin | + |
| N-Acetyl-D-Galactosamine | − | Glucuronamide | + | Tetrazolium Violet | + |
| N-Acetyl Neuraminic Acid | − | Mucic Acid | + | Tetrazolium Blue | + |
| α-D-Glucose | + | Quinic Acid | + | Nalidixic Acid | + |
| D-Mannose | w | D-Saccharic Acid | + | Lithium Chloride | + |
| D-Fructose | w | p-Hydroxy-Phenylacetic Acid | − | Potassium Tellurite | + |
| D-Galactose | w | Methyl Pyruvate | w | Aztreonam | w |
| 3-Methyl Glucose | − | D-Lactic Acid Methyl Ester | − | Sodium Butyrate | − |
| D-Fucose | w | L-Lactic Acid | + | Sodium Bromate | − |

* +: Positive; −: negative; w: weakly positive

**Table S2 Summary of functional annotations**

| Functional annotations | Number of Protein-coding genes (CDs) | Percentage (%) |
| --- | --- | --- |
| Total | 5,906 | 100 |
| COG | 3,453 | 58.47 |
| KEGG | 3,097 | 52.44 |
| GO | 3,572 | 60.48 |

**Table S3 Comparisons between the ANIs (ANIb and ANIm) of LWPZF and other related Pseudomonas**

| Strain | ANIb^a^ | ANIm^b^ |
| --- | --- | --- |
| WRS8 | 98.21 | 98.5 |
| KT2440 | 96.67 | 97.1 |
| Pf-5 | 79.03 | 84.48 |

^a^ ANI-blast, ^b^ ANI-MUMmer

**Table S4 Genes involved in plant growth promotion, heavy metal resistance, and biocontrol in the *P.* *putida* LWPZF genome**

| **LWPZF ORF ID (JNO42_)** | **Gene** | **Function** |
| --- | --- | --- |
| **IAA biosynthesis** |  |  |
| 27680 |  | tryptophan 2-monooxygenase (EC 1.13.12.3) |
| 09210 |  | phenylacetaldoxime dehydratase |
| 09225 |  | amidase (EC 3.5.1.4) |
| 09230, 09235 | *nthAB* | nitrile hydratase (EC 4.2.1.84) |
| **Phosphate solubilization** |  |  |
| 16315 |  | PQQ-dependent glucose dehydrogenase (EC 1.1.5.2) |
| 23645, 23640, 23635, 23630,  23625, 23620, 23615 | *pqqFABCDEG* | pyrroloquinoline quinone biosynthesis protein FABCDEG |
| 05140, 05145, 05150, 05155 | *pstSCAB* | phosphate ABC transporter substrate-binding protein PstS, permease subunit PstC, permease PstA, ATP-binding protein PstB |
| 21230 | *phoU* | phosphate transport system regulatory protein PhoU |
| 21250, 21245, 21240, 21235 | *pstSCAB* | phosphate ABC transporter substrate-binding protein PstS, permease subunit PstC, permease PstA, ATP-binding protein PstB |
| **ACC deaminase activity** |  |  |
| 13430 |  | 1-aminocyclopropane-1-carboxylate deaminase (EC 3.5.99.7) |
| **Siderophore production** |  |  |
| 01855 | *pvdS* | sigma factor |
| 01860 | *pvdL* | non-ribosomal peptide synthase for chromophore |
| 01945, 01950 | *pvdNM* | aminotransferase, dipeptidase |
| 01955 | *pvdP* | twin-arginine translocation signal domain-containing protein |
| 01960 | *fpvI* | sigma factor |
| 02110, 02115, 02120, 02125,  02140 |  | non-ribosomal peptide synthase |
| 02130 | *fpvA* | outer membrane ferripyoverdine receptor FpvA |
| 02135 | *pvdO* | formylglycine-generating enzyme PvdO |
| 02145 | *pvdE* | ATP-binding-cassette (ABC) transporter PvdE |
| 02150 |  | acetyltransferase |
| 02155 | *opmQ* | efflux transporter outer membrane subunit OpmQ |
| 02160 | *macB* | MacB family efflux pump subunit |
| 02165 | *pvdR* | efflux RND transporter periplasmic adaptor subunit pvdR |
| 04010 | *mbtH* | MbtH family protein |
| 04015 |  | thioesterase |
| 04030, 04035, 04040, 04045,  04050, 04055, 04060, 04065 |  | membrane proteins |
| 04070 | *pvdA* | L-ornithine hydroxylase |
| **Trehalose** |  |  |
| 02760 | *treS* | trehalose synthase |
| 02790 | *treY* | malto-oligosyltrehalose synthase |
| 02800 | *treZ* | malto-oligosyltrehalose trehalohydrolase |
| **Acetoin and 2,3-butanediol** |  |  |
| 07075 |  | acetolactate synthase large subunit |
| 17655 | *ilvN* | acetolactate synthase small subunit |
| 17660 |  | acetolactate synthase 3 large subunit |
| 27675 |  | acetolactate synthase large subunit |
| 24570 |  | acetoin dehydrogenase / butanediol dehydrogenase |
| **Tolerance against metal toxicity** | |  |
| 05450 | *arsH* | arsenical resistance protein ArsH |
| 05455 |  | arsenate reductase ArsC (EC 1.20.4.1) |
| 05460 |  | arsenic transporter |
| 05465 |  | ArsR/SmtB family transcription factor |
| 12680 | *cinQ* | NADPH-dependent 7-cyano-7-deazaguanine reductase |
| 12685 | *cinA* | cupredoxin-like copper-binding protein |
| 12690 | *cinR* | two-component system response regulator CinR |
| 12695 | *cinS* | two-component system sensor histidine kinase CinS |
| 21660, 24480 | *copB* | copper resistance protein B |
| 21670, 24490 | *copA* | copper resistance protein CopA |
| 21680, 28510 | *cusR* | HM response regulator transcription factor CusR |
| 21685, 28505 | *cusS* | HM sensor histidine kinase CusS |
| 21690 | *cusC* | HM efflux system outer membrane protein CusC |
| 21695 | *cusB* | copper/silver efflux system membrane fusion protein CusB |
| 21700 | *cusA* | copper/silver efflux system membrane protein CusA |
| 21705 | *cusF* | copper/silver efflux system periplasmic protein CusF |
| 28490 |  | chromate resistance protein |
| 10645 | *chrA* | chromate efflux transporter |
| 28495 | *chrA* | chromate efflux transporter |
| 11235 |  | cobalt-zinc-cadmium resistance protein CzcA |
| 11240 |  | cobalt-zinc-cadmium efflux system membrane fusion protein CzcB |
| 11245 |  | cobalt-zinc-cadmium efflux system outer membrane protein CzcC |
| 21780 |  | cobalt-zinc-cadmium resistance protein CzcD |
| 21860 |  | cobalt-zinc-cadmium resistance protein CzcA |
| 21865 |  | cobalt-zinc-cadmium efflux system membrane fusion protein CzcB |
| 21870 |  | cobalt-zinc-cadmium efflux system outer membrane protein CzcC |
| 28330, 28580, 29275 | *merF* | mercury resistance system transport protein MerF |
| 28335, 28430, 28585, 29270 | *merP* | mercury resistance system periplasmic binding protein MerP |
| 28340, 28425, 28590, 29265 | *merT* | Hg(II) transporter MerT |
| 28345, 28420, 28595, 29260 | *merR* | Hg(II)-responsive transcriptional regulator |
| 28565, 29290 | *merE* | broad-spectrum mercury transporter MerE |
| 28570, 29285 | *merD* | mercury resistance co-regulator MerD |
| 28575, 29280 | *merA* | mercury(II) reductase |
| **Biocontrol** |  |  |
| 01500 |  | PhzF family phenazine biosynthesis protein |
| 21190 |  | chorismate lyase (EC 4.1.3.40) |

**Table S5 Accession numbers of housekeeping genes used for phylogenetic tree construction**

|  | ***Pseudomonas putida* LWPZF** | ***Pseudomonas putida* BIRD-1** | ***Pseudomonas putida* KT2440** | ***Pseudomonas taiwanensis* WRS8** | ***Pseudomonas putida* GB-1** | ***Pseudomonas putida* E41** | ***Pseudomonas cremoricolorata* ND07** | ***Pseudomonas alkylphenolica* KL28** | ***Pseudomonas fluorescens* Pf-5** | ***Pseudomonas sp. CMR12a*** |
| --- | --- | --- | --- | --- | --- | --- | --- | --- | --- | --- |
| ***gyrB*** | JNO42_21615 | PPUBIRD1_0077 | PP_RS00065 | ICN73_RS06390 | PPUTGB1_RS00040 | CR511_RS00020 | LK03_RS06185 | PSAKL28_RS00055 | PFL_RS00020 | C4K39_RS00020 |
| ***rpoB*** | JNO42_24025 | PPUBIRD1_0484 | PP_RS02380 | ICN73_RS04245 | PPUTGB1_RS02445 | CR511_RS02525 | LK03_RS07660 | PSAKL28_RS02525 | PFL_RS28335 | C4K39_RS20135 |
| ***rpoD*** | JNO42_23685 | PPUBIRD1_0424 | PP_RS02050 | ICN73_RS04575 | PPUTGB1_RS02115 | CR511_RS02195 | LK03_RS07335 | PSAKL28_RS02175 | PFL_RS28710 | C4K39_RS19790 |
